# A Series of Essays on Inflammation and Its Varieties. Essay I. The Natural History of the Disease

**Published:** 1846-07

**Authors:** 


### A Series of Essays on Inflammation and its Varieties. Essay I. The Natural History of the Disease. By *H. Clutterbuck*, M.D. Octavo, pp. 67. London, 1846.

Of all that falls within the province of the practice of medicine, Inflammation always has obtained, and seems destined always to demand, the greatest share of attention. Its external and more obvious characters have long been minutely described, and are now well known, but its intimate nature, even with the aid of the microscope, is a matter of keen dispute, and affords a field for investigation that promises fertile results. Independently of this, the observation of disease has long taught most men the vast influence which it exerts in the production, course, and management of the various phenomena of morbid manifestations; and although the time has gone by when these were regarded as mere varieties of expression of the same essential and identical fact, yet many will yet agree with our author in affirming the proposition these Essays are intended to establish—"*That most diseases either consist in inflammation, or are consequences of it, more or less remote.*" Few have advocated this doctrine more boldly or more successfully than he has heretofore done in respect to a class of affections, for the explanation of which it would to most seem defective or faulty; and we are glad to find so veteran a writer again in the field, armed, we hope, with additional facts and arguments. Whether this is the case or not, however, we shall not yet have an opportunity of judging, as the present part of the work treats only of the natural history of the disease. The *characters* and *causes* of inflammation are lucidly stated, but we will confine ourselves to the section upon its *nature*. Dr. Clutterbuck maintains that the humoral pathology is in no wise competent to the explanation of the production of the phenomena of inflammation, and makes the somewhat obscure statement, that "a vitiated state of the *fluids*, when it does occur, is for the most part the *effect* of disordered action of the *solids;* and to be viewed rather in the light of an *exciting cause*, than as constituting the disease itself." It does not seem very clear how it can be both of these. In searching for the *proximate cause* in the solids, we must not limit our attention to the inspection of the effects of the disease on the dead, but likewise interrogate the phenomena in the living. Mr. Hunter and his followers maintain that the blood-vessels are more active in this state, and transmit the blood with greater force and velocity. They appeal to the comparative rapidity with which it flows from an inflamed part, and to the enlarged condition of the veins issuing from it—increased redness and augmented temperature being the natural consequences of these conditions. The sensibility and irritability of the part are increased, its movements more energetic, and its bulk augmented by increased secretion or new growth.

"Notwithstanding these strong, and, one would think, conclusive proofs of *increased activity* of the bloodvessels in *inflammation*, and incompatible as they seem to be with the notion of *debility*, there are, nevertheless, not a few who still maintain the opposite opinion—namely, that, in *inflammation*, the action of the vessels is weakened, instead of being increased, and the circulation in the part carried on more slowly and feebly than in health. The chief argument adduced on this occasion, seems to be derived from the enlargement of the vessels of the part; *distension* being considered as necessarily implying *weakness*. Thus, Dr. Billing observes, 'that the natural action of the arteries is *contraction*. Now, as the arteries in an *inflamed* part are larger than before, they must have contracted less, and consequently have acted with less force.' Another distinguished teacher and writer, of the present day (Dr. C. Williams), expresses himself to much the same effect. Speaking of *determination of blood* to a part, he denies that this is caused by *increased* action of the arteries, 'because,' he says, 'the only *active* property we know these vessels to possess is that of *slow* or *tonic contraction;* and this would *diminish* instead of *increasing* the motion and quantity of blood proceeding to the part.' 'The enlargement of vessels,' it is further said, 'where a determination of blood takes place, is effected by *arterial distension from behind,* acting on a tube that has already lost some of its contractile power. The arteries, thus enlarged, become channels for the conveyance of more blood, and with more force, into the *capillaries* and *veins* leading from them; these will become, in like manner, enlarged, and share the increase of force and motion thus supplied to them.' But if, as here alleged, the distended arteries of the part are in a *weakened* state, and the only active property of arteries is that of *slow* or *tonic* contraction,—whence, it might be asked, is derived that 'arterial distension from behind' which is said to produce the enlargement of the vessels of the inflamed part, seeing that the action of the heart is not at all increased in these cases? It is, besides, not easy to understand, how an enlarged tube, 'that has already lost some of its contractile power' in what is called *determination of blood,* should *thereby* become a channel for the conveyance of more blood, 'and with more force,' into the capillaries and veins leading from it, the contrary rather should be the case, according to ordinary hydraulic principles.

The notion, that the only active property possessed by arteries is that of '*slow* or *tonic* contraction,' leads to a denial of their having any share in the general circulation of the blood, this office being supposed to be performed by the heart alone; as, indeed, has been of late contended for also by Dr. Marshall Hall and a few other physiologists. The fallacy of this, however, was so clearly pointed out by Mr. Hunter, that one wonders so unfounded a supposition—one so inconsistent with striking facts—should still be held."

After adducing proofs that the arteries really possess an *oscillatory* action, *i. e.* a condition of alternate contraction and relaxation, Dr. Clutterbuck continues :—

"Such are the chief grounds that may be adduced in favour of the opinion entertained by Mr. Hunter and his numerous supporters, with regard to the *intrinsic* nature of *inflammation* considered as a *vital process;* viz. that it is a state of *increased activity*, chiefly observable in the *bloodvessels* of the part, these being the principal agents in effecting the various physical changes that are taking place. But while it is contended, that in *every inflammation* there is an increase of *vital activity* in the part affected, it is of great importance to bear in mind, that it is not simply a state of *increased action,* but an action of a preternatural and morbid kind, and subject to new laws, widely differing from those of health: as is proved by the results above stated. Of the *intrinsic* nature of this, however, we are, and probably ever shall remain, ignorant. We know it by observation alone, and by comparison with the healthy state. Its proper management, likewise as a matter of practice, is only to be learned in the same way, namely, by observation and experience. And even these are but uncertain and insufficient guides; because the disease is subject to be influenced by a variety of circumstances, many of which are unknown to us, and many beyond our ability to control. This acknowledgment of the imperfection of our art should inspire caution in the application of remedies; more especially, in the present day, when the *Materia Medica* consists, to so great an extent, of the most deleterious articles."

In his section on the *varieties* of inflammation, Dr. Clutterbuck characterizes many diseases as inflammatory not usually considered such, among which may be reckoned tic douloureux, ague, phthisis, &c. &c.; but we must reserve our objections to this summary procedure until we are in possession of the defence of his views in the future parts of the work.

The present part is a very able one, and fully maintains the reputation of its author.

